# Multi-Sensor Fault Diagnosis Based on Time Series in an Intelligent Mechanical System

**DOI:** 10.3390/s22249973

**Published:** 2022-12-17

**Authors:** Zhuoran Xu, Qianmu Li, Linfang Qian, Manyi Wang

**Affiliations:** 1 School of Computer Science and Engineering, Nanjing University of Science and Technology, Nanjing 210094, China; 2 School of Mechanical Engineering, Nanjing University of Science and Technology, Nanjing 210094, China

**Keywords:** intelligent mechanical system, multi-sensor, time series, fault diagnosis, Autoformer, transfer entropy

## Abstract

Intelligent mechanical systems are a focused area nowadays. One of the requirements of intelligent mechanical systems is to achieve intelligent fault diagnosis through the real-time acquisition and analysis of data from various sensors installed on mechanical components. In this paper, a new fault diagnosis method is proposed to solve the problems of difficulty in integrating the fault diagnosis algorithm and locating fault parts due to the complexity of modern mechanical systems. The complexity of modern industrial intelligent systems is due to the fact that the systems are composed of multiple components and there are various connections between them. Common fault diagnosis is to design specialized fault identification algorithms for the physical characteristics of each component, and the integration of different algorithms is a major challenge for system performance. Therefore, this paper investigates a general algorithm for the fault diagnosis of complex systems using the timing characteristics of sensors and transfer entropy. The fault diagnosis algorithm is based on the prediction of multi-dimensional long time series using Autoformer, and fault identification is performed based on the deviation of the predicted value from the actual value. After fault identification, a root cause analysis method of faults based on transfer entropy is proposed. The method can locate the component where the fault occurs more accurately based on the analysis of the cause–effect relationship of each component and help maintenance personnel to troubleshoot the fault.

## 1. Introduction

Machinery intelligence is the application of intelligent technology to machinery, with information technology, computer technology, control technology, and other forms of high technology into the construction of machinery to improve the stability, comfort, and operation of construction machinery and to improve work efficiency. Intelligent machines do not require the participation of operators. They acquire environmental information through the installation of various complex sensors so as to have self-perception, automatic control, autonomous health management, and other functions.

Traditional mechanical system health management methods, such as after-the-fact maintenance and regular monitoring, require a large number of human resources, financial resources, and time costs and cannot meet the requirements of being intelligent, intensive, and efficient. One of the basic responsibilities of autonomous health management is to monitor the number of important parameters and the working status of the main components of the machine in real time through the sensors installed in the key parts of the construction machinery. When the parameters are out of the normal range or in abnormal condition, the system performs condition monitoring and fault diagnosis. Artificial intelligence-driven fault diagnosis has become an important research component for emerging needs and innovations in industry. Ref. [1] proposed an early fault diagnosis method for rolling bearings based on noise-assisted signal feature enhancement and random resonance. The advantage of this algorithm is that it avoids the insufficiency of useful information filtering in traditional noise reduction methods and realizes the early diagnosis of mechanical equipment failures, which is of great significance to the smart factory. Ref. [2] proposed a multimodal fusion support vector classification method for gearbox faults. In deep-network diagnosis, ref. [3] used a sparse superposition self-encoder (SSAEs) for failure modes and crack sizes of bearings; ref. [4] combined wavelet packet energy and deep convolutional neural networks for the fault diagnosis of spindle bearing faults; ref. [5] developed a thermal image feature extraction method to achieve fault diagnosis for three induction motor faults; ref. [6] proposed a deep wavelet self-encoder and limit learning machine approach for the intelligent fault diagnosis of rolling bearings. Ref. [7] developed online diagnostics embedded in Industry 4.0 manufacturing systems applied to DES modelled by acyclic or reversible labelled Petri nets, which identify normal and faulty behavior of the system through verification based on a set of inequalities in the variables. Based on a new construction pattern recognition technique for health indicators, Ref. [8] proposed a general manufacturing multi-component fault detection and diagnosis method, which creates health indicators from a combination of meaningful features extracted from the time and frequency domains. These indicators can be used to detect the health of the system. Ref. [9] proposed a new ensemble sparse supervised model (ESSM) for mechanical fault diagnosis to improve the reliability of smart manufacturing. Ref. [10] applied digital twin technology to the fault diagnosis of rotating machinery, which integrates physical knowledge and data-driven intelligence into a model that enables accurate fault diagnosis and adaptive degradation analysis.

The above study shows that the existing fault diagnosis methods for intelligent machinery have two limitations: (1) Most multi-sensor diagnosis algorithms are target-specific diagnosis algorithms, such as bearings and rotating parts, and the algorithms are designed based on their physical characteristics. Therefore, these diagnostic algorithms have assumptions and limitations on their application scope. However, intelligent machines are usually complex systems with multiple devices with different functions. If different fault diagnosis algorithms are used for each device, it will complicate the system’s automatic fault diagnosis and algorithm integration. (2) In complex mechanical systems, the location of faulty components is a necessary measure to prevent the recurrence of mishaps. Especially when a new fault occurs, it is essential to analyze its cause to improve the overall system’s safety and quality. Current fault diagnosis algorithms are not good for the root cause analysis of multi-component systems, mainly because there are various connections between components, which causes problems in finding the root cause of a fault.

The main contributions of this paper are as follows:

- The previous idea of designing fault diagnosis algorithms based on special equipment characteristics is abandoned. Considering that the completion of tasks in industrial automation systems is usually time-based, the data from sensors have time series characteristics. Time series features can reflect the changes in industrial control processes, so we propose a fault diagnosis algorithm based on the time series features of intelligent mechanical system data. The algorithm is a fault diagnosis method applicable to multi-component complex mechanical systems, not only to one component;
- The fault diagnosis algorithm is divided into fault identification and root cause analysis. This paper adopts a fault identification algorithm suitable for multi-dimensional long-time series prediction to solve the prediction difficulties caused by many sampling data sources and high sampling accuracy in industrial control systems;
- After a certain fault is determined, a method for analyzing the fault cause of the multi-equipment system is designed to locate the fault location and help system maintenance personnel to eliminate the fault.

## 2. Related Work

In the research of multi-sensor fault diagnosis methods, most algorithms are designed for a specific target, and each algorithm has different assumptions and applicable objects. Therefore, various diagnostic algorithms have been proposed for multiple diagnostic targets in different fields.

### 2.1. Domain-Oriented Multi-Sensor Fault Diagnosis Methods

The existing multi-sensor fault diagnosis methods are divided into two categories: diagnosis methods based on signal analysis and fault pattern recognition.

#### 2.1.1. Fault Diagnosis Based on Signal Analysis

Diagnosis methods based on signal analysis usually use signal models, such as correlation functions, spectrum analysis, autoregressive moving averages, wavelet transforms, etc., to directly analyze measurable signals and extract symptom values such as the variance, amplitude, and frequency. The method can also use principal component analysis to study further the main symptoms of fault information, which can be used to reduce data in preparation for subsequent diagnosis.

Ref. [11] proposed a relevance vector machine (RVM) multi-sensor fault diagnosis method based on ant colony optimization (ACO) for gearbox fault detection. First, the collected sensor data is decomposed by EEMD, and 27 time-domain and frequency-domain features of the first three IMF components are extracted; then, distance evaluation technology is used for feature selection; finally, ACO-RVM is used for fault identification. Ref. [12] proposed a method based on wavelet correlation feature scale entropy and a self-organizing feature map (SOM) neural network for multi-sensor gear fault diagnosis in strong-noise scenarios. Ref. [13] proposed dual-tree complex wavelet transforms (DTCWT) to diagnose electrical and mechanical faults. Compared with traditional wavelet transforms, DTCWT-based feature extraction and fault isolation methods show a high performance. Ref. [14] uses the empirical mode decomposition method to extract the fault features of the vibration signal. It combines the ordered weighted average and fuzzy integration to fuse the multi-sensor information to obtain the fault diagnosis results of a wind turbine.

#### 2.1.2. Fault Pattern Recognition and Diagnosis Methods

The fault pattern recognition and diagnosis method is based on artificial intelligence and computer technology and is mainly used to judge the type and severity of the system faults.

Ref. [15] proposed a credit assignment-based fuzzy cerebellar model articulation controller (FCA-CMAC) neural network information fusion model. The model is an estimator for unknown continuous faults, and the proposed fault identification method can diagnose thrusters’ continuous, uncertain, and novel failure modes. The heading angle sensor (compass) signal, the yaw rate, and the control signal are used as the input of the FCA-CMAC, and the fault diagnosis results are obtained through offline training. Ref. [16] utilized the characteristics of parallel processing and the highly self-organizing and self-learning information of a fuzzy neural network to diagnose the diesel engine of an unmanned cabin, overcoming the limitation of a single system and obtaining better fault diagnosis results. Ref. [17] used fuzzy theory to establish the membership degree between the fault representation and the model, used a genetic algorithm to realize fault diagnosis, and applied it to the field of distribution networks. Ref. [18] designed a series of fault isolation observers based on radial-basis function neural networks to achieve the complete decoupling of faults in different aircraft parts, thus isolating and identifying multiple faults. Ref. [19] proposed a fault diagnosis strategy for induction motors based on support vector machine (SVM) multi-classifications. Fault features extracted from electrical and mechanical diagnostic media are inputted into the support vector machine, which performs feature data fusion. This data fusion capability dramatically improves the reliability of the proposed scheme compared to previous efforts in this field.

### 2.2. Non-Domain Multi-Fault Diagnosis Methods

Presently, research in fault diagnosis at home and abroad mainly focuses on specific equipment or methods. A few researchers have begun to study how to reduce the assumptions and limitations of the scope of application of the diagnostic algorithm and how to improve the fault diagnosis model to make the algorithm have a better adaptability and accuracy.

Mechanical automation systems have many types of equipment with different characteristics. If each piece of equipment adopts different fault diagnosis algorithms, it will cause significant difficulties for the system’s autonomous fault diagnosis and algorithm integrators. Therefore, it is vital to design a general or suitable fault diagnosis algorithm for most equipment.

To solve the NP-hard combinatorial optimization problem of dynamic multi-fault diagnoses (dMFD), Ref. [20] proposed a dMFD algorithm based on the successive Lagrangian relaxation and sub-gradient optimization method with backtracking to obtain the near-optimal performance of the dynamic multi-fault diagnosis. This method can significantly reduce the number of calculations. Ref. [21] studied the application of the maximum product algorithm (MPA) to the generalized multiple fault diagnosis (GMFD) problem. Ref. [22] discussed the application of dynamic ensemble coverage (DSC) to dynamic multiple fault diagnosis and how to infer the most probable temporal order of a parsimonious set of fault sources to account for the observed changes in the test results over time. Ref. [23] proposed a heuristic search algorithm based on quantum computing and evolutionary computing, the quantum excited competitive evolutionary algorithm (QuCEA), for the NP-hard problem of multi-fault diagnosis. The algorithm significantly improves the performance when dealing with matrix-intensive high-dimensional MFD problems. Ref. [24] addressed the ill-posed issue by incorporating a tight frame structure into the typical sparse learning model, which adaptively performs subspace identification and multi-source fault separation.

## 3. Core Idea

### 3.1. Problem Description

Due to the development of technology, industrial systems are becoming larger and larger, the number of components in the systems is increasing, and the systems’ complexity is increasing. Industrial control processes need to identify faults and analyze the causes to prevent the recurrence of errors and improve the safety and quality of the system. Fault diagnosis includes fault identification and root cause analysis.

Considering that the industrial control process is usually related to the time sequence and that the time sequence can reflect the operating state of the system, the industrial control process is generally associated with the time series. The time series can reflect the running state of the system. This paper takes the sensors distributed on each component as the data sources and, based on their time series characteristics, studies the fault diagnosis algorithm suitable for the complex system composed of multiple information sources.

**Time series**: In this paper, time series with the same time interval are abbreviated as:

$$E=\langle E_{1},E_{2},\ldots ,E_{n}\rangle$$

Time series data describe the changes in various equipment parameters in the work and contain the law and trend of the fault occurrences, which are an essential basis for fault detection in this paper. Therefore, issues sorted by time (fixed time interval) can be regarded as multi-dimensional time series data.

To perform fault diagnosis on a multi-dimensional time series, we need to solve the following problems, denoted as questions Q1–2:

Q1: In the process of industrial control, the sampling frequency of the sensor is usually in the microsecond level, so the time series formed by the sampling data of an information source is usually relatively long. Therefore, how to identify the faults in a long time series is the first problem to be solved;
Q2: Industrial automation systems are usually composed of multiple components, and there are various connections between the parts, so there is also a relationship between the sensor data. For example, if two components are running in sequence, once the first running component fails, the data for the latter running component will inevitably have problems. Therefore, when analyzing the cause of the failure, we must consider how to explore the relationship between the components to find the actual cause.

### 3.2. Overview of Our Model

**Figure 1:**
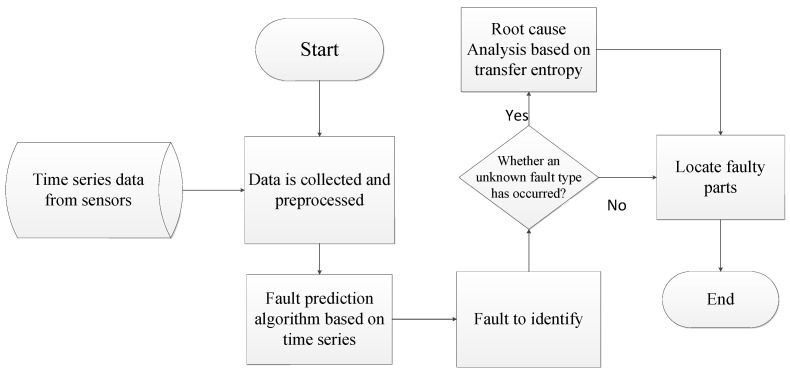
The process of the fault diagnosis method.

The process of multi-dimensional time series data fault identification and fault cause location are shown in Figure 1, which mainly includes three steps:

Step 1: Data collection. Collect system multi-dimensional time series data from sensors, and perform preliminary pre-processing for missing values, dead values, etc., to improve data quality.

Step 2: Anomaly detection. The long-time series prediction algorithm based on the Autoformer algorithm is used to identify the fault of the time series. At this time, normal data and fault data can be identified. When a new failure occurs, we do not know what caused it. The fault diagnosis algorithm cannot be answered, so go to Step 3 for root cause analysis.

Step 3: Root cause analysis. We believe system errors are often caused by the sudden change in one or more information sources. Therefore, we need to solve the problem of how to quickly find the sensor or combination of sensors that caused the failure. The algorithm performs root cause analysis by finding frequent abnormal data item sets and comparing the transfer entropy to screen and locate the abnormal components.

## 4. Fault Diagnosis Algorithm Based on Multi-Dimensional Time Series

### 4.1. Multi-Dimensional Time Series Fault Identification Based on the Autoformer Algorithm

The first step in fault diagnosis is to identify the fault. The idea adopted in this paper is to determine the occurrence of the faults by comparing the degree of deviation of the actual value of the time series to the predicted value. In industrial control, the sampling accuracy is usually at the microsecond level or even higher, so we can sometimes see that the sampling point of a control period is above 800. Many time series prediction algorithms, such as LSTM (long short-term memory) and Informer, cannot predict the future time series by remembering such a large amount of data. Therefore, this paper uses the Autoformer algorithm for long-time series predictions.

In long-output sequence predictions, Autoformer breaks through the traditional sequence decomposition as a pre-processing method and proposes a deep decomposition architecture. The method can decompose more predictable components from complex temporal patterns and proposes an autocorrelation mechanism based on stochastic process theory to achieve sequence-level connectivity, reduce complexity, and break the bottleneck of information utilization. Traditional time series decomposition refers to decomposing a time series into several components representing a class of potential time patterns, such as seasonal and trend-cyclical. Due to the future’s unknowability in forecasting problems, past sequences are usually decomposed and then forecasted separately. However, this results in predictions that are limited by decomposition effects and ignores future interactions between components. Autoformer decomposes the sequence as an internal unit and embeds it in the encoder–decoder. In the forecasting process, the model alternates between optimizing the forecasting results and decomposing the sequence, gradually separating the trend and the periodic terms from the latent variables to achieve progressive decomposition. Each component is gradually decomposed in the encoder based on the moving average idea, and each component is modelled separately in the decoder. Based on this progressive decomposition architecture, the model can gradually decompose the latent variables in the prediction process, obtain the prediction results of the cycle and trend components through the autocorrelation mechanism and accumulation method, and realize the alternating and mutual optimization of the decomposition and prediction results.

The multi-dimensional time series fault identification based on the Autoformer algorithm mainly consists of the following parts:

#### 4.1.1. Sequence Decomposition Module 

Time series decomposition usually decomposes the series into trend and seasonal terms and sometimes holidays and residuals. Autoformer’s series decomposition module is to decompose the series into trend $X_{t}$ and seasonal terms $X_{s}$:

(1) $$X_{t}=AvgPool\left(Padding\left(X\right)\right)$$

(2) $$X_{s}=X-X_{t}$$

The padding ensures that the sequence length remains unchanged, and AvgPool is a moving average. After obtaining the trend term, series − trend = season. The encoder input is a sequence of the length of I time points in the past $x_{en}\in {\mathbb{R}}^{I\times d}$. The decoder input includes the seasonal item $x_{des}\in {\mathbb{R}}^{\left(\frac{I}{2}+O\right)\times d}$ and the periodic item $x_{\det}\in {\mathbb{R}}^{\left(\frac{I}{2}+O\right)\times d}$. The variable *d* is the number of time series, and O is the length of time in the future.

#### 4.1.2. Encoder and Decoder 

The encoder focuses on modelling the seasonal part. The output is the past seasonal information, which is used as mutual information to help the decoder adjust the prediction results. Suppose we have N coding layers; the *i*-th coding layer is:

$${\left(x_{en}\right)}^{l}=Encoder{\left(x_{en}^{l}\right)}^{l-1}$$

$$s_{en}^{l,1}=SeriesDecomp\left(Auto-Correlation\left(x_{en}^{l-1}\right)+\left(x_{en}^{l-1}\right)\right)$$

(3) $$s_{en}^{l,2}=SeriesDecomp\left(FeedForward\left(s_{en}^{l-1}\right)+\left(s_{en}^{l-1}\right)\right)$$

Among them, FeedForward() is in the code: conv→relu→dropout→conv→dropout.

The decoder consists of two parts:

- The stacked autocorrelation mechanism of the seasonal components and the periodic nature of the sequences are used to aggregate subsequences with similar processes in different periods.
- An accumulation operation on the trend-cyclical component is used to gradually extract trend information from the predicted latent variables (the last one). Assuming we have M decoding layers, the internal details of the *i*-th decoding layer ${\left(X_{de}\right)}^{l}=Decoder\left(x_{de}^{l-1},x_{en}^{l}\right)$ are as follows:

$$s_{de}^{l,1},\tau_{de}^{l,1}=SeriesDecomp\left(Auto-Correlation\left(x_{de}^{l-1}\right)+\left(x_{de}^{l-1}\right)\right)$$

$$s_{de}^{l,2},\tau_{de}^{l,2}=SeriesDecomp\left(Auto-Correlation\left(s_{de}^{l,1},x_{en}^{N}\right)+\left(s_{de}^{l,1}\right)\right)$$

$$s_{de}^{l,3},\tau_{de}^{l,3}=SeriesDecomp\left(FeedForward\left(s_{de}^{l,2}\right)+\left(s_{de}^{l,2}\right)\right)$$

(4) $$\tau_{de}^{l}=\tau_{de}^{l-1}+w_{l,1}*\tau_{de}^{l,1}+w_{l,2}*\tau_{de}^{l}+w_{l,3}*\tau_{de}^{l}$$

#### 4.1.3. Autocorrelation Mechanism

Autoformer’s autocorrelation mechanism achieves efficient sequence-level connections to expand information. Generally, similar phases of different periods usually show similar subprocesses. Autoformer takes advantage of the inherent periodicity of the sequence to design an autocorrelation mechanism that includes period-based dependence and delayed information aggregation. 

Period-based dependency discovery employs stochastic process theory and computes correlations as confidence in unnormalized period estimates. Time delay aggregation is to aggregate similar subsequence information according to the calculated period length to realize the sequence-level connection.

The autocorrelation coefficient $R_{xx}\left(\tau \right)$ can be obtained using the fast Fourier transform (FFT) to find similar periodic subsequences:

$$s_{xx}\left(f\right)=F\left(x_{t}\right)F^{*}\left(x_{t}\right)=\int_{-\infty }^{\infty }x_{t}e^{-i2\pi tf}dt\int_{-\infty }^{\infty }x_{t}e^{-i2\pi tf}dt$$

(5) $$R_{xx}\left(\tau \right)=F^{-1}\left(S_{xx}\left(f\right)\right)=\int_{-\infty }^{\infty }S_{xx}\left(f\right)e^{i2\pi f\tau }df$$

where F and $F^{-1}$ represent the FFT and its inverse transform, respectively.

#### 4.1.4. Fault Identification Module 

The system can obtain the multi-dimensional time series forecast value in the next cycle according to the first three steps. The abnormal judgment of the time series is determined according to the degree in which the actual value deviates from the predicted value (called the forecast deviation). A threshold value is set in the algorithm, and when the prediction deviation of any time series exceeds the threshold value, it can be determined that the system has failed.

$$\delta^{i}={\left(y_{pre}\right)}^{i}-{\left(y_{act}\right)}^{i}$$

(6) $$s^{2}=\frac{\sum_{i=1}^{n}\left({\left(\delta^{{}_{i}}-\delta \right)}^{2}\right)}{n}$$

$y_{act}$ is the actual value, $y_{pre}$ is the predicted value, and δ is the average value of this set.

### 4.2. Failure Root Cause Analysis 

System fault diagnosis not only needs to focus on the superficial phenomenon of the fault, but also on gradually finding the root cause of the problem and solving it. Especially for a complex system composed of multiple components, it is vital to find the fault location when the fault occurs. The root cause analysis approach adopted in this article assumes that when a component fails, it is reflected in the values of one or more sensors.

Classical root cause analysis methods, such as Adtributor, iDice, and HotSpot, are commonly used in O&M analysis to find the indicators that lead to abnormal business occurrences. However, there are two problems in the application of these analysis methods in mechanical multi-sensor fault diagnosis. First, most of the O&M indicator values are discrete, while the sensor values are continuously transformed, so the algorithm for determining the abnormal indicators is relatively complex; second, there are causal relationships between some subsystems in mechanical systems, and none of the existing algorithms can judge the causal relationships between indicators well. For the above two problems, we designed a root cause analysis method based on transfer entropy.

In the fault identification and location of industrial automation systems based on multi-sensors, we call the sensor record an issue at each time point in the operation process.

**Figure 2:**
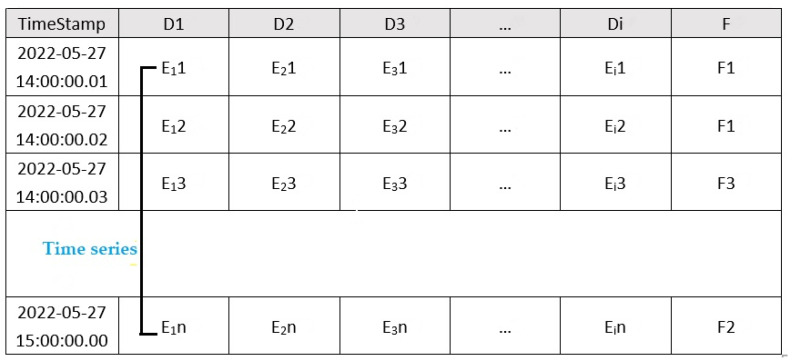
The structure of the issue and multi-dimensional time series.

Issue: The issue consists of multiple parts: timestamps, attribute D, and fault code F. A typical issue can include multiple attributes, each corresponding to a specific sensor. The sensors can be different kinds of sensors, such as speed sensors, displacement sensors, etc. Each sensor has its attribute value E, which can be considered time series data (time series). A typical issue contains various sources of information (called attributes), which can be of different kinds, such as motors, encoders, etc. The structure is shown in Figure 2:

According to the above analysis, the outliers of the faulty part or the part affected by it should frequently appear in the fault record, so we need to identify the attribute or attribute combination whose value is abnormal in the fault issue. Therefore, identifying attribute combinations is presented here as a pattern mining problem: the goal is to search for an attribute combination that isolates the entire multi-dimensional time series dataset into two parts: the fault state and the normal state. The algorithm is related to time series and needs to check whether the data under collection conforms to the time series characteristics. This means that the combination of attributes must be effective. In addition to being influential, the effective combination should also be related to emerging problems.

Summarizing the above analysis, the combinations of attributes that cause system errors should meet the following elements:

- For each failure, the set of elements should be able to explain the error as much as possible;
- For each failure, the set of elements should conform to Occam’s razor and should be as concise as possible in form;
- Of all the dimensions, the most unexpected dimension and the element where the true and expected values differ most should be found.

The specific process of the algorithm is as follows:

(1) Filter the abnormal attribute collection:

The effective combination we are looking for should be related to emerging failures. In other words, it is necessary to find the effective combination corresponding to the occurrence of the failure. The abnormal attribute is found according to Formula (6). The abnormal attribute item in the collected data group at each time point is marked as 1, and the normal attribute item is marked as 0.

(2) Calculate frequent item sets (Algorithm 1):

According to the previous analysis, it is necessary to identify the frequently occurring attribute value sets (frequent item sets) from the issue. This paper only considers the many combinations in fault events for computational efficiency. The reports are directly pruned and deleted for combinations that are not large enough. We do this by using data mining algorithms, calculating support, and setting thresholds.
**Algorithm 1:** Calculate frequent item sets algorithm. **Input:** A transaction database D  A minimum support threshold S  An optional parameter N indicates the maximum length an itemset could reach;  **Output:** frequent item sets L;
**initialize**k*← 1*$L_{k}$**← {1-itemsets that satisfy minimum support S }//**find the set L1 of frequent 1-itemsets**while**$L_{k}$**≠ Ø***if*∃N∨(∃N∧k<N)$C_{k+1}$*← candidate itemsets generated from*$L_{k}$**for each transaction t in database D do***increment the counts of *$C_{k+1}$*contained in t*$L_{k+1}$**← candidates in**$C_{k+1}$**that satisfy minimum support S**k**←**k**←1***return*$U_{kL_{k}}$**end for****end for**

According to the above analysis of attribute combination elements that lead to system errors, we can choose the candidate set *C* containing the most attributes as the possible root cause set

$$C_{}=\left[\begin{array}{cccc} D_{1} & D_{2} & \cdots & D_{k} \end{array}\right]$$

(3) Isolation power-based pruning:

Considering the causality between some attribute items, it is necessary to further eliminate the possible redundancy in the result set to determine the fault point further. In this paper, causality analysis based on transfer entropy is adopted.

Transfer entropy is a method based on probability distribution, information entropy, and statistics to find the causality between time series. Since the length of the time series needed is large, transfer entropy can only be used in neural signals and electroencephalograms in the era of small general data volumes. With the application of various sensors, transfer entropy plays an essential role in revealing the correlations of sensing data.

Transfer entropy [25] is based on the assumption that acquiring information about causes reduces the uncertainty of our observations. Transfer entropy studies the transfer of information between variables and can calculate how much the information transfer can reduce the uncertainty of the observed system. When the transfer entropy of *X* to *Y* is greater than *Y* to *Y*, we call *X* the cause and *Y* the effect and use this to establish a causal relationship between the two variables. It has been proven that transfer entropy can not only be applied to nonlinear time series, but can also be sensitive to granger causality.

Transfer entropy is an index to measure the directional transmission of the information of two time series, and $TE_{X\to Y}$ represents the amount of information transferred from *X* to *Y*. Given two time series, $X=\left\{x_{1},x_{2,\ldots },x_{T}\right\}$ and $Y=\left\{y_{1},y_{2,\ldots },y_{T}\right\}$, where *X* is the length of the time series and $x_{1}$ and $y_{1}$ are, respectively, the first observed values, and so on, the following formulas can be obtained:

(7) $$TE_{Y\to X}=\underset{x_{n+\tau },x_{n},y_{n}}{\sum }p\left(x_{n+\tau },x_{n},y_{n}\right)lb\left(\frac{p\left(x_{n+\tau },x_{n},y_{n}\right)p\left(x_{n}\right)}{p\left(x_{n},y_{n}\right)p\left(x_{n+\tau },x_{n}\right)}\right)$$

(8) $$TE_{X\to Y}=\underset{y_{n+\tau },x_{n},y_{n}}{\sum }p\left(y_{n+\tau },x_{n},y_{n}\right)lb\left(\frac{p\left(y_{n+\tau },x_{n},y_{n}\right)p\left(y_{n}\right)}{p\left(x_{n},y_{n}\right)p\left(y_{n+\tau },y_{n}\right)}\right)$$

where n is the discrete-time index, τ is the prediction time, and p represents the probability distribution.

According to Formulas (7) and (8), the transfer entropy matrix T is obtained by calculating the transfer entropy between all pairs of the attribute items.

$$T=\left[\begin{array}{cccc} t_{11} & t_{12} & \cdots & t_{1n}\\ t_{21} & t_{22} & \cdots & t_{2n}\\ \vdots & \vdots & \ddots & \vdots \\ t_{n1} & t_{n2} & \cdots & t_{nn} \end{array}\right]$$

The transfer entropy value between the two attribute items in the frequent item set is compared in T. The attribute item with higher transfer entropy is the cause, while the attribute item with lower transfer entropy is the result.

If $t_{ij}>t_{ji}$, $D_{i}$ is the cause, and $D_{j}$ is the result. In the fault cause analysis, we consider the abnormal $D_{j}$ value to be caused by the abnormality $D_{i}$, so we do not consider $D_{j}$ it to be the cause of the fault.

## 5. Experimental Results and Analysis

### 5.1. Data Set 

**Figure 3:**
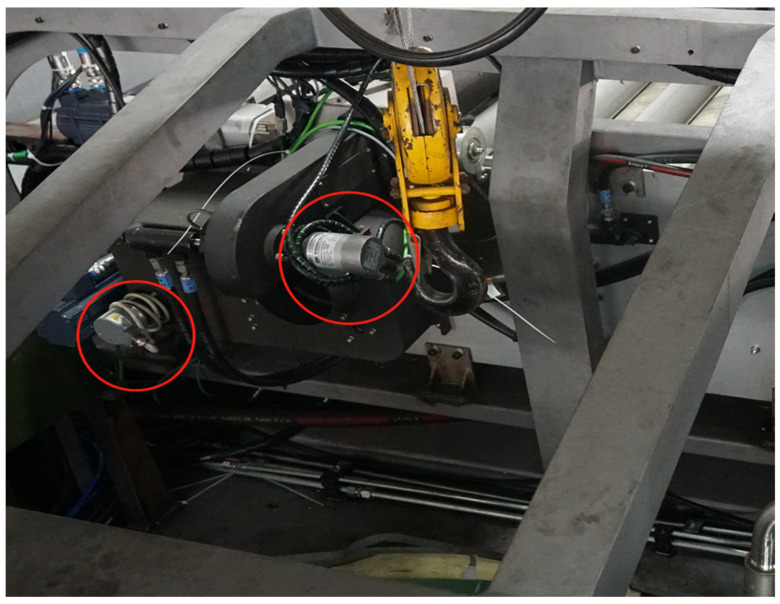
Schematic diagram of the sensor installation for the drug delivery mechanism.

The experimental data were obtained from the drug delivery subsystem of a special piece of equipment. The subsystem consists of the following components: the flip part (D1), the coordinating component (D2), the main drug supply component (D3), the second drug supply part (D4), the rotation of the second drug supply part (D5), and the main drug supply rotation mechanism (D6). The flip and coordination components are responsible for moving the drug delivery tubes to the designated position, the main and second drug supply components are responsible for drug delivery, and the rotation components of the main and second drug supply components are responsible for moving them to the designated position. The data come from the encoders and inductive sensors deployed on each component. A partial view of the system is shown in Figure 3 (the encoder is marked with a red circle in the diagram):

The data format is shown in Table 1.In this table, data are collected at 1 ms intervals. D1–D5 use absolute encoders, which record the current displacement value of the component, and D6 uses inductive sensors, which represent the status of the four positions of the main drug supply rotation mechanism.

**Figure 4:**
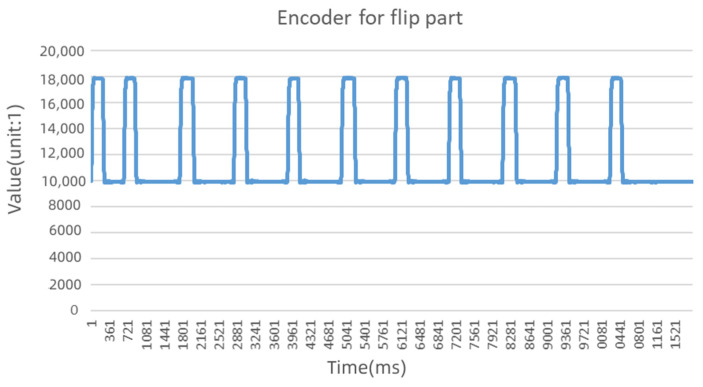
The time series of the flip mechanism.

Taking the information source D1 as an example (the encoder of the flip part), it can be seen in Figure 4 that the value of the encoder has obvious timing characteristics, and the running process of the part can be seen from the time series.

As seen from the above example, the time series data of the industrial control system reflects obvious periodicity, and the number of sampling points in each cycle is 800–900. In the final stage of the experiment, the component malfunctioned and did not perform as expected.

### 5.2. Experimental Results of the Multi-Dimensional Time Series Fault Identification

**Figure 5:**
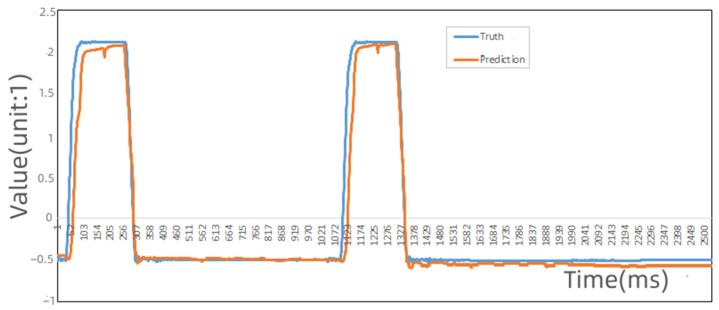
The predicted and actual values of information source D1 using the Informer algorithm.

**Figure 6:**
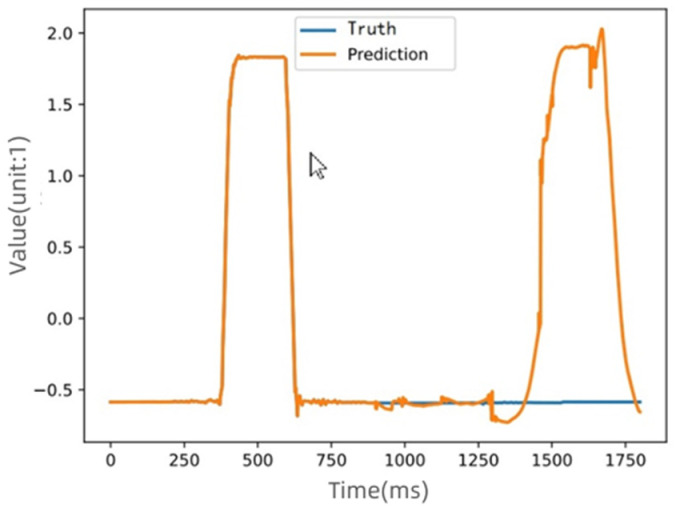
The predicted and actual values of information source D1 using the Autoformer algorithm.

The prediction results with the Informer and Autoformer algorithms are shown in Figure 5 and Figure 6:

Figure 5 is the comparison of the predicted values and the actual values before and after the failure of D1 (the value of the encoder is the value obtained after normalization). It can be seen that since the algorithm could not memorize a long-term sequence well, the prediction result could not be accurately obtained.

Figure 6 is the same D1 data using Informer to predict the results (the value of the encoder is the value obtained after normalization). We can see that the actual values of this part deviate greatly from the predicted values. The Autoformer algorithm predicts the D1 value of the system in the correct state.

According to the algorithm, the predicted values and actual values of D1 can be obtained (the values are the results of standardized processing) as shown in Table 2:

According to Formula (6), the forecast deviation value S of each information source is shown in Table 3.

As seen from the table, when the threshold value is set to 0.5, the D1, D2, and D6 values exceed the threshold, so it can be determined that the system is faulty.

### 5.3. Experimental Results of the Multi-Dimensional Sequence Root Cause Determination

#### 5.3.1. Calculate the Transfer Entropy Matrix T

According to Formulas (7) and (8), the transfer entropy *T* was calculated as:

$$T=\left[\begin{array}{cccccc} 0 & 1.8644 & 1.6721 & 1.6652 & 1.7594 & 1.7139\\ 1.8644 & 0 & 1.6721 & 1.6652 & 1.7594 & 1.7139\\ 1.6343 & 1.6343 & 0 & 4.6498 & 5.0793 & 4.8836\\ 1.6140 & 1.6140 & 4.5843 & 0 & 4.7244 & 4.5319\\ 1.7110 & 1.7110 & 5.0867 & 4.7384 & 0 & 5.1411\\ 1.6274 & 1.6274 & 4.8915 & 4.5470 & 5.1519 & 0 \end{array}\right]$$

#### 5.3.2. Fault Location and Cause Analysis

According to Algorithm 1, we set the threshold to 0.5, min_support = 0.8, min_confidence = 1, and we obtained the set of fault-related attributes as shown in Table 4:

According to the above experimental results, the frequent item set *C* was obtained:

$$C=\left[\begin{array}{ccc} D_{1} & D_{2} & D_{6} \end{array}\right]$$

From the matrix *T*, we obtained:

$$t_{12}=t_{21},\;t_{16}>t_{61},\;t_{26}>t_{62}$$

**Figure 7:**
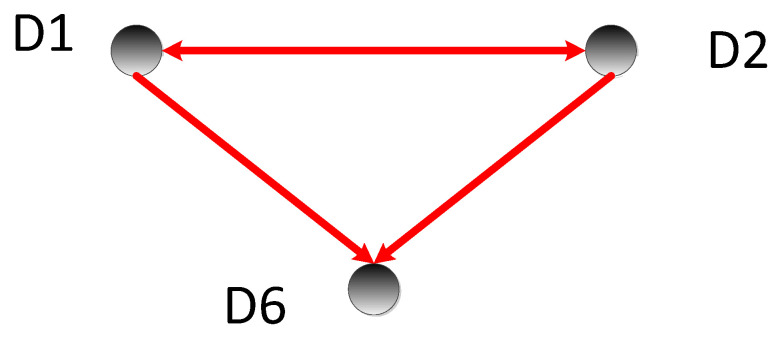
Cause-and-effect diagram 1.

By comparing the transfer entropy values, we obtained the causal relationship between nodes, as shown in Figure 7; the direction of the arrows represents the causal relationship.

It can be inferred that D1 and D2 are causal to each other, D1 is the cause of D6, and D2 is the cause of D6. The value of D6 is affected by D1 and D2, so D6 was deleted when analyzing the cause of the fault. Through the above analysis, the fault location was finally located in D1 and D2. During the actual repair process, it was found that the reason for the error in this experiment was “the drug delivery tube was not at the flip termination point and therefore the drug delivery was prohibited”.

Taking another set of data as an example, we obtained the set of fault-related attributes as shown in Table 5:

The results mined with the frequent item sets are $C=\left[\begin{array}{ccc} D_{3} & D_{4} & D_{5} \end{array}\right]$. From the matrix *T*, we obtained: $t_{34}>t_{43}$, $t_{35}<t_{53}$, $t_{45}<t_{54}$.

**Figure 8:**
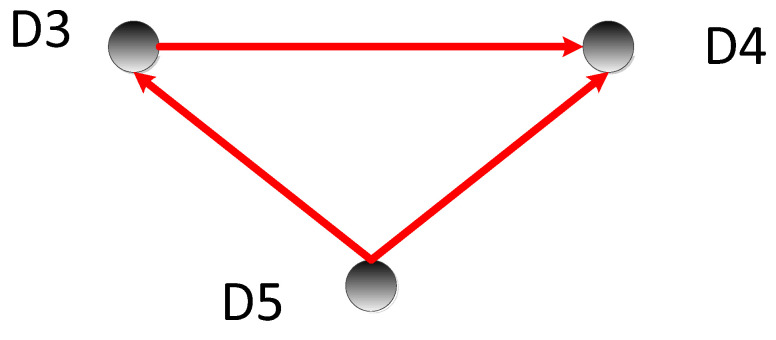
Cause-and-effect diagram 2.

By comparing the transfer entropy values, we obtained the causal relationship between nodes, as shown in Figure 8; the direction of the arrows represents the causal relationship.

It can be inferred that D3 is the cause of D6, and D5 is the cause of D3 and D4. The values of D3 and D4 are affected by D5. Through the above analysis, the fault location was finally located in D5. During the actual repair process, it was found that the reason for the error in this experiment was that the “pushing mechanism prohibits rotary action”.

## 6. Conclusions

The complexity of a mechanical system composed of multiple components is reflected in two aspects: first, in their different types of components and their different physical characteristics, and second, in the interaction between the components. This paper focuses on how to overcome the problems brought by complexity in mechanical system fault diagnosis. The specific research directions were as follows:

- In order to overcome the fact that the integration of different fault diagnosis algorithms would bring suffering to the system performance due to the variety of each component, this article studied fault diagnosis algorithms that could be generalized for different components. In this article, the time series behavior of components was studied, and the time series-based fault diagnosis algorithm was used to replace the diagnosis algorithm based on the physical characteristics of components. In this paper, we adopted a multidimensional time series fault identification algorithm based on the Autoformer algorithm, which can better solve the prediction problem of multidimensional long-term series.
- Because multiple components often affect each other, they can bring uncertainty to the analysis of the cause of failure. To address this problem, our second research direction was to analyze the component relationships for a better root cause analysis of faults. In this paper, we designed a transfer entropy-based root cause analysis method, which can identify the causal relationships between components and thus more accurately uncover the causes of failures.
- The current fault prediction algorithm can be further improved in the prediction of long time series and in the optimization of the root cause analysis algorithm based on transfer entropy, such as through the pruning strategy, which is also the direction of future research.

## Figures and Tables

**Table 1:** The structure of the experimental data.

Timestamp	D1	D2	D3	D4	D5	D6	F
1	9941	1,320,661	159,902,125	168,834,489	437,400	1001	0
2	10,002	1,320,722	159,902,125	168,834,489	437,400	1001	0
3	10,088	1,320,808	159,902,125	168,834,489	437,400	1001	0
4	10,209	1,320,929	159,902,125	168,834,489	437,400	1001	0
5	10,341	1,321,061	159,902,125	168,834,489	437,400	1001	0
6	10,501	1,321,221	159,902,125	168,834,489	437,400	1001	0
7	10,687	1,321,407	159,902,125	168,834,489	437,400	1001	0
8	10,900	1,321,620	159,902,125	168,834,489	437,400	1001	0
9	11,124	1,321,844	159,902,125	168,834,489	437,400	1001	0
10	11,381	1,322,101	159,902,125	168,834,489	437,400	1001	0
11	11,657	1,322,377	159,902,125	168,834,489	437,400	1001	0
12	11,945	1,322,665	159,902,125	168,834,489	437,400	1001	0
13	12,247	1,322,967	159,902,125	168,834,489	437,400	1001	0
14	12,541	1,323,261	159,902,125	168,834,489	437,400	1001	0
15	12,828	1,323,548	159,902,125	168,834,489	437,400	1001	0
16	13,107	1,323,827	159,902,125	168,834,489	437,400	1001	0
17	13,377	1,324,097	159,902,125	168,834,489	437,400	1001	0
18	13,659	1,324,379	159,902,125	168,834,489	437,400	1001	0
……							
11,440	9906	1,320,626	158,435,308	166,383,448	437,364	1001	402
11,441	9906	1,320,626	158,435,308	166,383,448	437,364	1001	402
11,442	9906	1,320,626	158,435,308	166,383,448	437,364	1001	402
11,443	9906	1,320,626	158,435,308	166,383,448	437,364	1001	402
11,444	9906	1,320,626	158,435,308	166,383,448	437,364	1001	402
11,445	9906	1,320,626	158,435,308	166,383,448	437,364	1001	402
11,446	9906	1,320,626	158,435,308	166,383,448	437,364	1001	402
11,447	9906	1,320,626	158,435,308	166,383,448	437,364	1001	402
11,448	9906	1,320,626	158,435,308	166,383,448	437,364	1001	402
11,449	9906	1,320,626	158,435,308	166,383,448	437,364	1001	402
11,450	9906	1,320,626	158,435,308	166,383,448	437,364	1001	402
11,451	9906	1,320,626	158,435,308	166,383,448	437,364	1001	402
……							

**Table 2:** The predicted values and actual values of D1.

Prediction	Truth	δ
−0.56658614	−0.59247607	0.02588993
−0.5630873	−0.59267557	0.02958827
−0.901269	−0.5918989	−0.3093701
−0.8979537	−0.59138376	−0.30656994
−0.901961	−0.5911302	−0.3108308
−0.9009461	−0.5908066	−0.3101395
−0.90133065	−0.5907445	−0.31058615
−0.90918547	−0.59061235	−0.31857312
−0.9073556	−0.5904101	−0.3169455
−0.89864874	−0.59022665	−0.30842209
−0.8890775	−0.59076995	−0.29830755
−0.8896758	−0.5911186	−0.2985572
−0.8773504	−0.5912724	−0.286078
……		
0.017941706	−0.5902468	0.608188506
0.025903054	−0.5902228	0.616125854
0.033776082	−0.5901876	0.623963682
0.039482415	−0.5901555	0.629637915
0.044889383	−0.59027636	0.635165743
0.04916533	−0.59035623	0.63952156
0.87285906	−0.5903951	1.46325416
0.44322592	−0.590445	1.03367092
0.50573945	−0.5904538	1.09619325
0.73909134	−0.59047365	1.32956499
0.05072336	−0.59050447	0.64122783
0.5027535	−0.59053236	1.09328586
……		

**Table 3:** The forecast deviation value S of each information source.

Sensor	D1	D2	D3	D4	D5	D6
*S*	1.236	0.73	0.012	0.012	0.006	0.898

**Table 4:** The frequent item sets of the system.

Frequent Item Sets	Number of Occurrences
D6	866
D1	846
D2	844
D1, D2	841
D1, D6	813
D2, D6	811
D1, D2, D6	808

**Table 5:** The frequent item sets of the system (example 2).

Frequent Item Sets	Number of Occurrences
D3	1357
D4	1357
D5	1357
D3, D4	1357
D3, D5	1357
D4, D5	1357
D3, D4, D5	1357

## Data Availability

The data presented in this study are available from the corresponding authors. The data cannot be made public as it relates to ongoing projects.
